# Ethiopian Medicinal Plants Used for Respiratory Tract Disorders: Ethnomedicinal Review

**DOI:** 10.1155/2023/7612804

**Published:** 2023-01-10

**Authors:** Alemtshay Teka, Melesse Maryo

**Affiliations:** ^1^Endod and Other Medicinal Plants Research Unit, Aklilu Lemma Institute Pathobiology, Addis Ababa University, Addis Ababa, P.O.B. 1176, Ethiopia; ^2^Ethiopian Biodiversity Institute Addis Ababa, Home Based in Kotebe Meteropolitan University, Department of Biology, Addis Ababa, P.O.B. 30726, Ethiopia

## Abstract

Respiratory tract infections (RTIs) refer to infections in any part of the respiratory tract, which are common, with most of the world's population contracting at least one infection annually. These infections are becoming important causes of death and morbidity due to the rapid development of antimicrobial resistance that has resulted in reduced efficacy of existing drugs. Different local societies residing in Ethiopia have been reported to use traditional medicinal plants to treat RTIs. Nevertheless, up-to-date summarized data on the diversity of plants used in the traditional medicine system to treat RTIs in Ethiopia are lacking. The purpose of this review was to assess plant species used in traditional medicine to treat respiratory tract infections in Ethiopia. It attempts to compile available data required for undertaking further scientific investigations. The data were collected by searching for published scientific articles and other grey literature. Following this, medicinal plant (MP) diversity, growth forms, plant parts used, modes of remedy preparation and application, sources and distributions, and frequently treated respiratory disorders were examined. An Excel spreadsheet and SigmaPlot software were used to summarize and present the data. Two hundred twenty-nine (229) plant species that have been used to treat respiratory disorders in Ethiopia were documented. Lamiaceae was the most cited family (27 species), followed by Asteraceae (23 species), and Fabaceae (18 species), whereas cough was primarily cited as being treated by MPs and scored the highest frequency of citation (FOC = 243), followed by the common cold (FOC = 151) and asthma (FOC = 63). The top-cited plant species used in the treatment of RTIs were *Eucalyptus globulus* (6.8%), *Allium sativum* (5.5%), *Zingiber officinale* (4.2%), *Ruta chalepensis* (3.8%), and *Ocimum lamiifolium* (2.8%). Herbs were the dominant plant growth form (46%) used to treat respiratory diseases, and the most commonly used MP parts were leaves (37%). The leading traditional method used for preparation was decoction (25.5%), and the remedies were usually administered orally (64.6%). The MP origin reported was mainly from the wild (59%). High diversity of medicinal plants was reported as being used to treat various RTIs in Ethiopia. Information obtained from this review could be used as a reference for the selection of plants for further pharmacological, phytochemical, and toxicological investigations for their possible therapeutic applications and the development of new plant-based drugs.

## 1. Introduction

Respiratory tract infections (RTIs) refer to infections in any part of the respiratory tract that could affect the nasal passages, the bronchi, and the lungs [1, 2]. They are common, with most of the world's population contracting at least one infection annually [1]. The respiratory tract is highly susceptible to infections due to its continuous exposure to the gaseous environment including particulate organic material, such as bacteria and viruses. In order to reduce such risk of respiratory tract infections, the human body has developed a range of strategies including filtering out in the nasal hairs, inertial impaction with mucus-covered surfaces in the posterior nasopharynx and more. In spite of the different strategies developed to defend microorganisms from reaching the lower respiratory tract, the frequency of infection occurrences in the respiratory tract poses serious problems due to their high prevalence with associated significant mortality rates and economic loss [3, 4]. Respiratory conditions include bronchitis, common cold, pharyngitis, sinusitis and influenza. Coughing, sneezing, fever, shortness of breath, sore throat, and headache are the common signs associated with respiratory tract disease [2].

Traditional medicine has been used for thousands of years [5], and contemporary allopathic medicine is embedded in it. It was indicated that 65–80% of the world's healthcare practice involves the use of traditional medicine [6]. Likewise, in Ethiopia, its application as a health care system has a long history, and about 80% of the population is dependent on traditional medicine [7–9]. It has been used to treat disease and reduce numerous symptoms without any scientific proof of efficacy [10]. Cultural acceptability, economic affordability, and efficacy against certain types of diseases compared to modern medicine probably have made traditional medicine a preferable health care system in Ethiopia as well as in other developing countries [7, 8, 11, 12]. Moreover, despite the paucity of scientific evidence associated with the efficacy and safety of traditional medicine, the increasing preference for traditional medicinal use is supported by the promising results local people gain from using traditional medicinal plants in the treatment of various diseases [13, 14].

Plant-based traditional medicines are widely used in several indigenous societies. Various kinds of herbal remedies have evolved from diverse cultures and geographical regions. Thus, every kind of traditional herbal medicinal system has its own exclusive way of understanding and treating a disease [6]. These remedies are progressively becoming more prevalent in modern society as substitutes for synthetic medicines [15] and have been renowned as a source of raw materials for the commercial production of currently utilized antimicrobial agents. Additionally, the isolation of bioactive compounds and pharmaceutical drugs derived from nature and traditional remedies has produced promising results [6, 16]. Besides, in the search for the development of new potent drugs and to overcome resistance to contemporary pharmaceuticals, using medicinal plants as a source of new bioactive compounds is highly recognized [11].

Respiratory tract infection is a common and significant cause of illness and death around the world. Globally, more than one billion people suffer from respiratory tract infection, which makes it one of the leading causes of death in developing countries [17, 18]. The rapid development of resistance to the drugs currently in use is one of the major challenges in containing diseases of the respiratory tract [18]. Traditionally used medicinal plants had been a viable resolution in managing such diseases [4]. In Ethiopia, respiratory disease is reported among the commonest illnesses for which self-medication has been taken [13]. However, medicinal plants (MPs) used in the traditional healthcare system are often undocumented and are at risk of being lost [19]. In addition, up-to-date summarized data on the diversity of plants used in the traditional medicine system to treat RTIs in Ethiopia is lacking. Therefore, this review is intended to compile herbals that were reported in Ethiopia to treat and control symptoms and disease of the respiratory tract. It also attempts to compile available data required for undertaking further scientific investigations. Accordingly, plants that are reported as being used in the Ethiopian Flora area to treat signs or diseases, including asthma, breathing difficulties, bronchitis, common cold, cough, flu, influenza, pneumonia, sinusitis, whooping cough, lung infection, lung abscess, respiratory tract problems, and pharyngitis, were reviewed in this paper. The electronic database research, ethnobotanical books, and grey literature were accessed from the Internet and libraries in Ethiopia.

## 2. Methods

This review was carried out following the recommendations stated in the Preferred Reporting Items for Systematic Reviews and Meta-Analyses (PRISMA) statement [20].

### 2.1. Search Strategy

Medicinal plants that have been used to treat respiratory tract disease in the Ethiopian traditional health care system were collected from published research articles, academic theses, and books. Terms and phrases like “ethnobotanical study,” “traditional use of plants,”, “medicinal plants”, “ethnopharmacological study,” “respiratory tract infection,” “respiratory disease/disorder,” and “Ethiopia” were used to search freely available journals in various electronic databases (including Google, Google scholar, Scopus, Web of Science). Addis Ababa University institutional repository (AAU-IR) was used to access the academic theses. The ethnobotanical books were accessed from the Ethiopian Biodiversity Institute library, and important grey literature was consulted.

### 2.2. Study Selection

#### 2.2.1. Inclusion Criteria

Openly available published research articles, academic theses, books, and some grey literature written in English on MPs used to treat human ailments in Ethiopia were reviewed. Ethnomedicinal documents released from the 1900s to the latest (January 2022) published articles without restriction in the methodology were included. Medicinal plants identified to species level with full ethnomedicinal information and reported as being used to treat respiratory disease or related signs including asthma, breathing difficulties, bronchitis, common cold, cough, flu, influenza, pneumonia, sinus, whooping cough, lung infection, lung abscess, respiratory tract problems, pharyngitis, in the flora of Ethiopia were incorporated and summarized in this study.

#### 2.2.2. Exclusion Criteria

Studies that use only the local name of the plant or identify it only at the genus level were excluded. In addition, articles about ethnoveterinary studies and review papers were disregarded.

**Figure 1:**
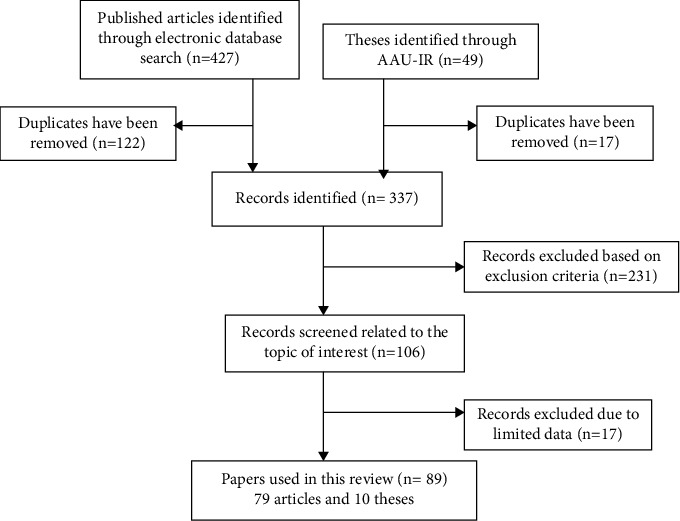
Flow diagram showing the selection strategy of the reviewed papers.

Following the search strategy and inclusion criteria described above, a systematic review of 89 studies and a book on documented ethnomedicinal knowledge used to treat respiratory disease in different parts of Ethiopia was undertaken (Figure 1).

### 2.3. Data Collection

Data on Ethiopian MPs used traditionally to treat respiratory disease signs were retrieved and summarized. Data including scientific names, local names (if available), growth form, habitat, plant part used, preparation method, and route of administration were extracted. Missing information on family names, growth forms, and verified accepted scientific names were checked and retrieved using The Flora of Ethiopia and Eritrea Volumes and https://www.tropicos.org website.

### 2.4. Data Analysis

The data was tabulated and summarized using an Excel spreadsheet. SigmaPlot software was used for analyzing the data using descriptive statistics (percentage, frequency of citation) and for drawing the graphs.

## 3. Results and Discussion

### 3.1. Medicinal Plants Diversity

**Figure 2:**
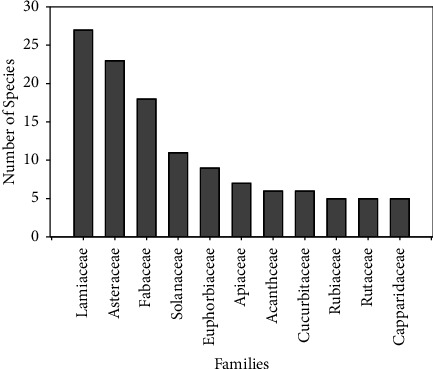
Plant families based on the number of species used to treat respiratory diseases (each with greater than four species).

A total of 229 plant species are traditionally used for the treatment of respiratory system disorders in Ethiopia (Annex 1). The plant species are distributed in 168 genera and 74 families. Few families (11) represent a higher percentage (53%) of the species reported. Moreover, the families Lamiaceae, Asteraceae, Fabaceae, and Solanaceae were represented by more than ten species (Figure 2). These families were represented by 12, 10, 8, and 5% of the total species identified (229) for respiratory treatment in Ethiopia. Several studies reported the dominance of these families of MPs in Ethiopia [21–23] and in ethnomedicinal uses [7, 14]. The dominant uses of these families for the treatment of respiratory disorders were also reported in studies from various other countries. The greater representation of Asteraceae, Fabaceae, and Lamiaceae is consistent with the study from South Africa [1, 24], which is linked with the higher number of species in these families. The dominance of Family Lamiaceae is consistent with the use of medicinal plants for respiratory diseases in Pakistan [12], Fabaceae and Rutaceae in Kenya [19], and Asteraceae in South Africa [1]. The use of MPs in the control of diseases that belong to selected families could be influenced by the culture and availability of plant species in a geographical location [25]. It could also likely be due to a wider range of distribution as well as the production of prospective secondary metabolites with potent antimicrobial and anti-inflammatory activity [26, 27].

### 3.2. Frequently Used Medicinal Plants

**Figure 3:**
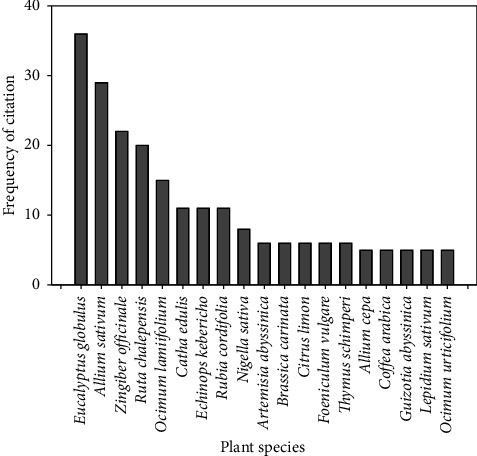
The most frequently used plant species used to treat respiratory disease in Ethiopia.

The plant species that represented the highest number of citations were *Eucalyptus globulus* (36 citations), *Allium sativum* (29 citations), *Zingiber officinale* (22 citations), *Ruta chalepensis* (20 citations), and *Ocimum lamiifolium* (15 citations). *Catha edulis, Echinops kebericho*, and *Rubia cordifolia* each had eleven citations. The other eleven species also had five to eight citations (Figure 3). Similar to the present study, the use of *A. sativum, E. globulus, Nigella sativa, R. cordifolia*, and *Z. officinale* for the treatment of respiratory disease was reported in Africa [28].

The traditional use of *Eucalyptus* species has been reported in other African countries as Tunisian folk medicine from which the essential oil is used to treat respiratory tract problems including bronchitis, pharyngitis, and sinusitis [29]. The antimicrobial activity tests of the essential oil of *E. globulus* showed strong antimicrobial activity, especially against *Streptococcus pyogenes, Staphylococcus aureus, Acinetobacter baumannii*, and *Klebsiella pneumonia* [30]. These bacteria are causative agents of respiratory tract infection in humans and are resistant to antibiotics. Moreover, eucalyptus oil exerts a protective effect against lipopolysaccharide (LPS) plus *Klebsiella pneumonia* induced lung injury [31].

*Allium sativum* has been traditionally used to treat respiratory diseases in South America (e.g., Brazil), North America (Mexico), Europe (Spain and France), and Asia (Pakistan and Turkey) [32], showing its most common use in different countries. Active ingredients such as ferulic acid, flavonoids, anthocyanins, gallic acid, protocatechuic acid, and others were identified in *A. sativum* [32]. Besides Ethiopia and other developing countries, *Z. officinale* is also reported as being used in folk medicine from SE Asia and inGreco-Roman traditions in Brazil, Australia, Africa, China, India, Japan, Bangladesh, Taiwan, Mexico, the Middle East, Jamaica, and parts of the United States grow the rhizomes for its medicinal purpose [33]. *Z. officinale* was cited as the dominant medicinal plant for the treatment of respiratory tract disease in Pakistan [12] and Kenya [19], respectively. Nutraceutical compounds of *Z. officinale* claimed to have medicinal value include gingerols and zingerone. Of these, gingerols are thought to be the most pharmacologically active components [34]. It has been reported as an active inhibitor of *Mycobacterium avium* [35] and shows antibacterial effects on *S. aureus,* which is a causative agent for respiratory disorders [36]. These scientific facts corroborate the indigenous use of selected medicinal plant species in the treatment of respiratory problems across the globe.

### 3.3. Growth Forms

**Figure 4:**
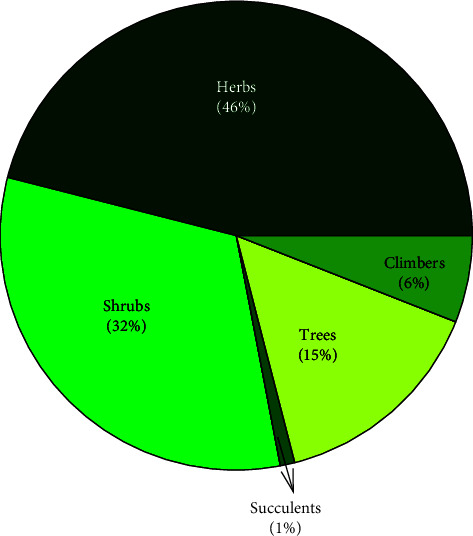
Proportion of growth forms of the medicinal plants.

The dominant growth forms of plants used for the treatment of respiratory diseases in Ethiopia were herbs (46%), followed by shrubs (32%) (Figure 4). Several previous reports have indicated the dominance of herbs in the traditional plant-based health care systems in Ethiopia [8, 9, 37–39], Pakistan [12], and South Africa [1]. The dominance of herbs could be due to their accessibility, perceived efficacy, and the sociocultural beliefs and practices of the healers in treating an ailment with herbs [40–42]. Thomas et al. [43] also indicated a higher possibility of obtaining pharmacologically active compounds in herbs as compared to the other growth forms. In addition, as herbaceous plant growth rates are comparably fast, the dominant use of herbs is considered beneficial for the sustainable production of medicinal plants [1].

### 3.4. Plant Parts Used

**Figure 5:**
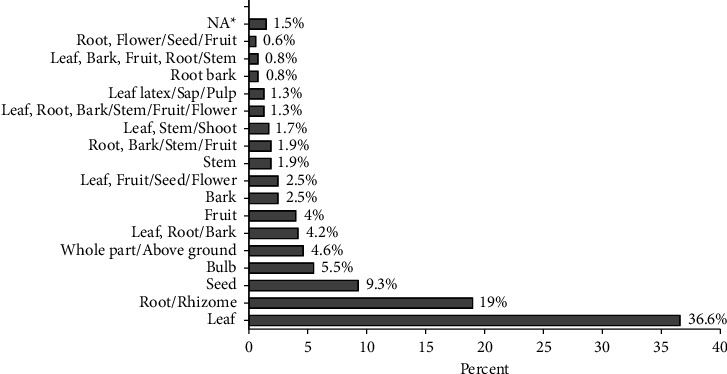
Medicinal plant parts used and their proportion of citation.

The most commonly used medicinal plant parts to treat respiratory tract ailments were leaves (37%), followed by root/rhizome (19%), and seed (9.3%) (Figure 5). Generally, the remedial preparations involve both single plant parts (79%), and more than one parts (21%) of the medicinal plant species. The dominant use of leaves in the ethnomedicinal plant use systems in Ethiopia [39, 40, 44–47] and specifically for treating respiratory disorders were reported in different studies [1, 12, 48]. Such preference could be associated with ease of harvest or preparation and the synthesis of abundant active constituents (bioactive ingredients) in the leaves which are pharmacologically active against certain diseases [40, 46, 49, 50]. It could also be attributed to the renewal potential of leaves that does not put medicinal plants at risk of extinction over a period [12, 49]. Despite the dominance of leaves, it is reasonable to consider bioactivity and pharmacological investigation of more plant parts of the suggested medicinal plant, as the probability of identification of bioactive compounds might be increased.

### 3.5. Modes of Remedy Preparation and Application

Medicinal plant species used to treat respiratory disease in Ethiopia are traditionally prepared using about 18 different methods. The most commonly used traditional method for preparation is decoction, which constitutes about 26%, followed by decoction and inhaling (8%) (Table 1). A similar method of remedy preparation was reported from the investigation of ethnomedicinal studies in various parts of Ethiopia [7, 51, 52]. Likewise, the decoction was reported as a dominant means of the preparation of medicinal plant remedies for respiratory diseases in South Africa [1], Pakistan [12], and Kenya [19]. The preference of a decoction over other traditional methods of remedy preparation could be related to the long established experience of the local community and the tested efficacy of particular plant medicines. However, during this review process, about 21% of the citations missed assigning methods of preparation.

### 3.6. Routes of Administration

The largest proportion of traditional herbal remedies used to treat respiratory diseases are taken orally (65%), followed by nasal (9%) and inhale/steam bath/fumigation (8.8%) (Table 2). Similar findings were reported from different studies of ethnomedicinal plants in Ethiopia [7–9, 39, 40, 42, 46] and other east African countries [19]. This could be associated with the dominance of internal ailments' treatment in areas of traditional herbal medicine use [40] and respiratory disorders.

### 3.7. Sources of Medicinal Plant Species

**Figure 6:**
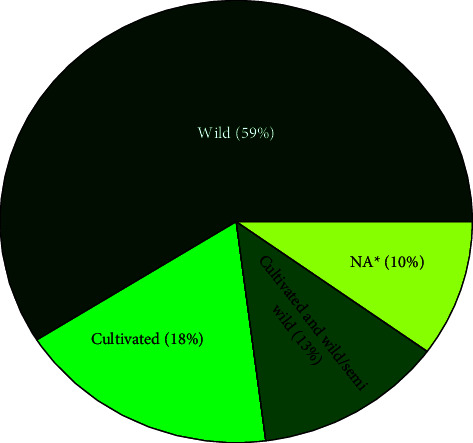
Proportion of sources of MP species used for the treatment of respiratory diseases (^*∗*^NA means not available; sources were not indicated in the reviewed papers).

About fifty-nine percent of the MP species used to treat respiratory disease are obtained from the wild (59%), followed by cultivated areas (18%) (Figure 6). Likewise, several ethnomedicinal studies in Ethiopia [38, 40, 45, 46] and other east African countries [19], indicate that the main sources of MPs are wild vegetation. Nevertheless, many of the wild habitats are experiencing great threats of destruction caused due to deforestation, agricultural expansion, urbanization, investment, and road construction, which impede MPs' sustainability and call for urgent conservation measures [42, 47].

### 3.8. Frequently Treated Respiratory Tract Disorders

**Figure 7:**
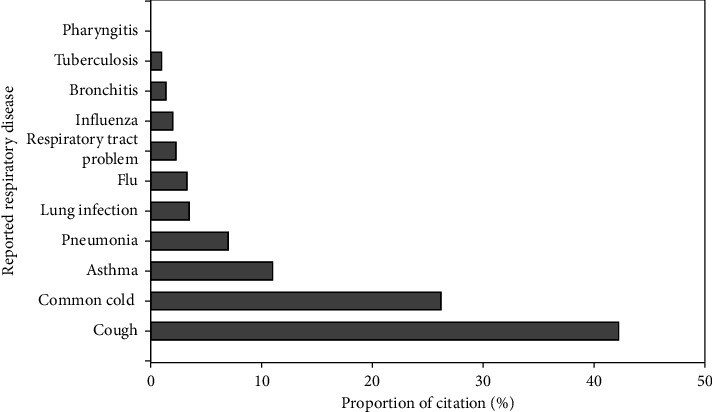
Relative proportion of respiratory disease treated by medicinal plants in Ethiopia (RT indicates respiratory tract; some MPs were cited in the reviewed articles generally for RT problems).

The majority of documented plant species are used as medicine to treat cough (42.2%), common cold (26.2%), and asthma (11%) (Figure 7). Similarly, studies from Kenya [19] and Pakistan [12] showed that the major respiratory disease to which most traditional medicinal plants applied was cough, showing its prevalence as a respiratory problem across many countries. The richness of remedies could be also associated with the prevalence of the disease.

### 3.9. Distribution of the Medicinal Plants

**Figure 8:**
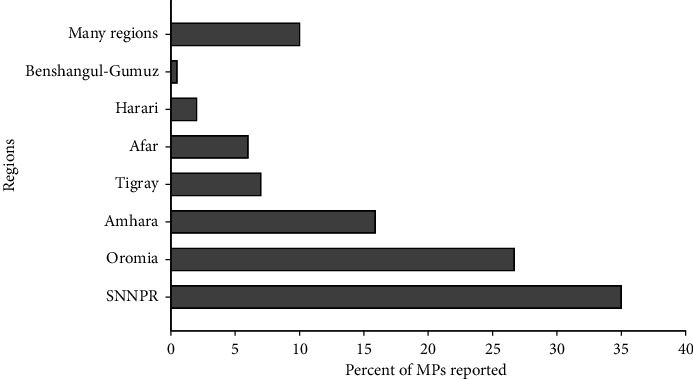
Proportion of reported medicinal plants used to treat respiratory disease across the regional states in Ethiopia.

The majority of the medicinal plants used to treat respiratory diseases were reported from the Southern Nations Nationalities and Peoples Regional State/SNNPR (35%) and Oromia (26.7%). This accounts for two-thirds (62%) of the total medicinal plant records used to treat respiratory disease (Figure 8). This has to do with the role of traditional knowledge that is associated with the number of cultures and traditions in which SNNPR is the home for the largest number of ethnic groups (i.e., 70%) in Ethiopia, and Oromia is the largest. Besides, both regions are known to be among the most populous regions in the country [53, 54]. Likewise, another study also reported the presence of higher medicinal plant diversity in the two regions [55]. The botanical diversity of the MP species might have been also linked with its varied agroclimatic conditions, diversity of the soil, topography, tradition of people's link with nature, cultural knowledge that is associated with the physical environment, and the higher number of ethnomedicinal studies that have been conducted in the regions. With respect to distribution, most of the medicinal plants documented have a wider ecological distribution in different regions of Ethiopia.

## 4. Conclusions and Recommendations

Ethiopia possesses rich biocultural diversity, specifically in terms of traditional knowledge of medicinal plants used to treat respiratory diseases. The community in different parts of the country uses highly diversified medicinal plants (229) to treat respiratory tract disorders and related signs. The species diversity may indicate the existence of respiratory tract disease as a common problem in the country. This diversity is not well exploited and documented for its population benefit. Thus, plants with a higher frequency of citation are recommended for further pharmacological, phytochemical, and toxicological investigations for their possible therapeutic applications and for developing new plant-based drugs.

## Figures and Tables

**Table 1:** Mode of remedy preparation and relative frequency of citation.

Mode of preparation	Frequency of citation	%
Decoction	121	25.5
Decoction and inhaling	37	7.8
Crushed and pounded	35	7.4
Fumigating	30	6.3
Crushed, homogenized with cold water	25	5.3
Concoction	20	4.2
Chewing	14	3
Crushed and pounded/concoction	14	2.9
Rub the part and sniff	13	2.7
Extracting the juice/oil/latex/	12	2.5
Chopping and chewing	11	2.3
Making infusion	11	2.3
Crushed and sniffed	9	1.9
Cooking	8	1.7
Crushed/decoction	4	0.8
Decoction and fumigating	4	0.8
Chewing, spitting	3	0.6
Decoction; concoction	3	0.6
NA (not available)	101	21.3

Total	475	100

**Table 2:** Routes of traditional herbal administration and relative frequency of citation.

Routes of administration	Frequency of citation	%
Oral	307	64.6
Nasal	43	9.1
Inhale/steam bath/fumigation	42	8.8
Oral, nasal/inhale/	21	4.4
External/dermal/topical/	12	2.5
Oral, dermal	3	0.6
Nasal, steam/inhale	3	0.6
Oral, nasal, dermal	8	1.7
Chewing, spitting	1	0.2
NA (not available)	35	7.4

Total	475	100.0

## Data Availability

All data generated or analyzed during this study are included in this published article.

## Supplementary Materials

Supplementary Materials Additional file 1: list of reviewed traditional medicinal plants used to treat respiratory disease in Ethiopia. The additional file shows the local names, growth form, part used, sources of the medicinal plant, modes of preparation, and application.
