# Supplementary material for: Ethiopian Medicinal Plants Used for Respiratory Tract Disorders: Ethnomedicinal Review
Source: Evid Based Complement Alternat Med. 2023 Jan 10;2023:7612804. doi: 10.1155/2023/7612804 (PMC9845041; doi:10.1155/2023/7612804)
Supplement: Supplementary Materials — Additional file 1: list of reviewed traditional medicinal plants used to treat respiratory disease in Ethiopia. The additional file shows the local names, growth form, part used, sources of the medicinal plant, modes of preparation, and application. [file 7612804.f1.doc]

**Additional file 1**: List of reviewed traditional medicinal plants used to treat respiratory disease in Ethiopia.

**Growth form** **(GF):** Herb-H, Shrub-S, Tree-T, Climber-Cl, Succulent-Su: **Local name:** Am-Amharic, Or-Oromifa,T-Tigrigna, Ge- Gedeoffa, G- Guragigna, Ha- Hadiyigna, Ka-Kafficho, Kam- Kambattissa, K-Kebena, Ko-Koorete, Kon-Konta, Ma- Maale, Me-Meinit language, Sh- Shinasha, Si-Sidama; **Part used (PU)**: Bark-B, Fruit-Fr, Flower-Fl, Leaf-L, Leaf latex-La, Stem-St, Root bark-RB, Seed-S, Seed oil-SO, Bulb-Bu, Rhizome-Rh, Whole part-WP, Shoot-Sh, **Mode of Application -** MOA: **Mode of Preparation** **(MOP):** 1-Decoction, 2-Decoction and inhaling/fumigation, 3-Crushed & pounded, 4-Crush, homogenize with cold water/Infusion, 5-Fumigating, 6-Concoction, 7-Crush/Rub and sniff, 8-Extracting the juice/oil/latex/, 9-Chopping, chewing, 10-Cooking, 11-Crushed and Concoction/decoction, 12-Chewing, spitting: **Habitat**: Wild-W, Cultivated-C, Wild and Cultivated-WC: **FC**- Frequency of citation: ‘NA/-’_Not stated: ‘Bold ****’ - Endemic**.

| **No.** | **Scientific name [Family]** | **GF** | **Local name** | **Traditional Use (FC)** | **MOA** | **MOP** | **Habitat** | **FC** | **References** |  |
| --- | --- | --- | --- | --- | --- | --- | --- | --- | --- | --- |
| 1 | *Abutilon figarianum* Guill. & Perro [Malvaceae] | H | - | Flu | Nasal | 9 | W | 1 | [56] |  |
| 2 | *Acacia albida* Del. [Fabaceae] | T | Gerbi (Or) | Cough, Pneumonia | Oral | 6 | W | 1 | [57] |  |
| 3 | *Acacia mellifera* (M. Vahl) Benth. [Fabaceae] | S | - | Flue | Nasal, Oral | - | W | 1 | [56] |  |
| 4 | *Acacia nilotica* (L.) Willd ex Del [Fabaceae] | T | - | Cough | Oral | 3 | W | 1 | [58] |  |
| 5 | *Acacia oerfota* (Forssk.) Schweinf. [Fabaceae] | S | - | Flue, coughing | Oral | - | W | 1 | [56] |  |
| 6 | *Acalypha fruticosa* Forssk. [Euphorbiaceae] | S | - | Lung Infection | Inhale | - | W | 1 | [56] |  |
| 7 | *Acalypha indica* L. [Euphorbiaceae] | S | - | Lung Infection | Oral | - | W | 1 | [56] |  |
| 8 | *Achyranthes aspera* L. [Amaranthaceae] | S | Nole (Si) | Cough | Oral | 1 | W | 1 | [59] |  |
| 9 | *Acmella caulirhiza* Del. [Asteraceae] | H | Shishimo (Ka) | Cough | NA | - | W | 1 | [60] |  |
| 10 | *Adhatoda schimperiana* ( Hocst.) Nees [Acanthaceae] | S | Sensel (Am) | Asthma | Oral | 1 | W | 1 | [58] |  |
| 11 | *Aframomum korrorima* (Braua.) Jansen. [Lamiaceae] | H | Wokkaasha (Ha) | Cough | Oral | 9 | C | 1 | [46] |  |
| 12 | Ajuga alba (Gurke) Robyni [Lamiaceae] | H | Anamuro(Or) | Common cold | Oral | 3 | WC | 1 | [51] |  |
| 13 | *Ajuga integrifolia* Ham. Buch [Lamiaceae] | H | Harmagusa (Am) | Cough, Common cold | NA | 1 | WC | 1 | [58], (64) |  |
| 14 | *Albizia amara* (Roxb.) B. Boivin [Fabaceae] | T | Ondoddee (Ko) | Cough | Oral | 3 | W | 1 | [61] |  |
| 15 | *Albizia malacophylla* (A. Rich) Walp. var. *ugandensis* Bak.f. [Fabaceae] | T | Jebiya (Sh) | Cough | Oral | - | W | 1 | [8] |  |
| 16 | *Allium cepa* L. [Alliaceae] | H | Chele Dukusho (Ka) | Cough, Asthma(2), Whooping cough, Common cold | Oral | 9 | C | 4 | [46], [60], [65], [66] |  |
| 17 | *Allium sativum* L. [Alliaceae] | H | Nech shinkurt (Am), Tuma (Gu) | Cough (11), Asthma (2), Common cold (7), Influenza (2), Pneumonia (4), Infection in upper respiratory tract, Lung abscess | Oral, Nasal | 1,2,4,6,7,9 | C | 19 | [44], [67], [68] |  |
| 18 | *Aloe macrocarpa* Tod. [Aloaceae] | H | Geneno’o (Ha), Ret (Am) | Cough | Oral | 4 | W | 1 | [46] |  |
| 19 | ***Aloe pubescens* Reynolds ** [Aloaceae]** | Su | Hargessa (Am) | Cold /Qilensa | NA | - | W | 1 | [63] |  |
| 20 | ***Aloe pulcherrima* Gilbert and Sebsebe ** [Aloaceae]** | Su | Sete- Iret (A) | Asthma | Oral | 1 | W | 2 | [49], [70] |  |
| 21 | *Andrachne aspera* Spreng. [Euphorbiaceae] | H | Etse-Tekeze (A) | Asthma | Oral | 4 | W | 2 | [58] [49] |  |
| 22 | *Anethum foeniculum* L. [Apiaceae] | H | Insilal (Am) | Cough | Nasal | 5 | C | 1 | [58] |  |
| 23 | *Anethum graveolens* L. [Apiaceae] | H | Mechwelago (Ka) | Cough | NA | - | W | 1 | [60] |  |
| 24 | *Anogeissus leiocarpa* (DC.) Guill. & Perr. [Combretaceae] | T | Hanse (Ti) | Pneumonia | Oral | 4 | W | 1 | [62] |  |
| 25 | *Artemisia abyssinica* Sch. Bip. ex A. Rich. [Asteraceae] | H | Tiroo (Or), Chikugne (Am) | Cough (4), Pneumonia | Oral | 1,4 | WC | 3 | [14], [37], [60], [71] |  |
| 26 | *Artemisia afra* Jack. ex Willd. [Asteraceae] | H | Aemechoae (Ko), Chikugn (Am) | Common cold, Cough, Pneumonia | Oral, Inhale | 1,3,7 | WC | 4 | [37], [46], [72] |  |
| 27 | *Artemisia absinthium* L. [Asteraceae] | H | Aguffa (Or), Natrar (Gu), Ariitti (Am) | Cough (3) | Oral | - | WC | 3 | [58], [39], [73] |  |
| 28 | *Asparagus africanus* Lam. [Asparagaceae] | H | Yet'ec'ufo(Sh), Yeset Kest (Am) | Common cold, Cough (2) | Oral | 1 | WC | 3 | [68], [74] |  |
| 29 | *Asparagus racemosus* Willd. [Asparagaceae] | S | Yet'ec'ufo(Sh) | Common cold | Oral | 1 | W | 1 | [68] |  |
| 30 | *Asparagus setaceus* (Kunth) Jessop [Asparagaceae] | S | Yet'ec'ufo(Sh) | Common cold | Oral | 1 | W | 1 | [68] |  |
| 31 | *Azadirachta indica* A. Juss. [Meliaceae] | T | Nim (Am) | Cough | Oral, Nasal | 2 | WC | 1 | [69] |  |
| 32 | *Balanites aegyptiaca* (L.) Del. [Balanitaceae] | T/S | Bedeno (Or), Got, Kutan (S) | Influenza, Lung Infection, Flu, cough | Oral, Nasal | 1 | W | 2 | [56], [57] |  |
| 33 | *Balanites rotundifolia* (van Tiegn.) Blatter [Balanitaceae] | S | _ | Flue, cough | Nasal, oral | - | W | 1 | [56] |  |
| 34 | *Barleria eranthemoides* R. Br. [Acanthaceae] | H | Shishi (Or) | Cold/ Ka fatime | NA | - | W | 1 | [63] |  |
| 35 | *Becium filamentosum* (Forssk.) [Lamiaceae] | S | _ | Lung Infection | Oral | - | W | 1 | [56] |  |
| 36 | *Bersama abyssinica* Fresen [Meliantaceae] | S | Jejjebba (Ge), Teberako (Sd) | Bronchitis (2), Cough | Oral | 4,11,12 | W | 3 | [52] , [75], [77] |  |
| 37 | *Bidens ghedoensis* Mesfin **** [Asteraceae] | H | Heedoormaaxa (Ha) | Cough, pneumonia | Oral | 4 | WC | 1 | [46] |  |
| 38 | *Boscia coriacea* Pax. [Capparidaceae] | S | _ | Lung Infection | Oral | - | W | 1 | [56] |  |
| 39 | *Brassica carinata* A. Braun [Brassicaceae] | H | Gomenzeera, Goommana (Or), Kolpo (Maale language) | Common cold | Oral | 4 | C | 4 | [38], [79], [80], [82] |  |
| 40 | *Brassica juncea* (L.) Czern. & Coss. [Brassicaceae] | H | Sannaafiya (Kon) | Pneumonia | Oral | 4 | WC | 1 | [37] |  |
| 41 | *Brassica nigra* (L.) Koch [Brassicaceae] | H | Shanafo (Ka), Sanafica (Am) | Cough, Common cold | Oral | - | C | 2 | [60], [79] |  |
| 42 | *Buddleja polystachya* Fresen. [Loganiaceae] | T | Bulshaana (Ha) | Cough, pneumonia | Oral | 1 | WC | 1 | [46] |  |
| 43 | *Cadaba farinosa* Forssk. [Capparidaceae] | S | _ | Lung Infection | Nasal | - | W | 1 | [56] |  |
| 44 | *Cadaba glandulosa* Forssk. [Capparidaceae] | S | _ | Lung Infection | Oral | - | W | 1 | [56] |  |
| 45 | *Capparis tomentosa* Lam. [Capparidiaceae] | S | Andiel (Ti) | Common cold | Oral | 1 | W | 1 | [62] |  |
| 46 | *Capsicum annum* L. [Solanaceae] | H | Chilli | Respiratory diseases | Nasal | - | C | 1 | [58] |  |
| 47 | *Carica papaya* L. [Caricaceae] | T | Papaya (Am) | Pneumonia, Cough (2), Lung disease (Dhibe somba) | Oral, Nasal | 1,2,4 | C | 3 | [51], [83], [84] |  |
| 48 | *Carthamus tinctorius* L. [Asteraceae] | H | Yeahya suf (Am) | Commnon cold, Cough | Oral | 1 | W | 2 | [76], [78] |  |
| 49 | *Cassipourea malosana* (Baker) Alston [Rhizophoraceae] | S | Lookoo (Am) | Pneumonia | NA | - | W | 1 | [87] |  |
| 50 | *Catha edulis* (Vahl) Forssk ex Endl. [Celastraceae] | S/T | Chat (Amh), Chele Chato (Ka) | Cough (4), Common cold (2), Asthma | Oral | 1,3 | C | 6 | [58], [38], [60], [66], [79], [87] |  |
| 51 | *Celosia polystachia* (Forssk.) C.C. Towns. [Amaranthaceae] | H | _ | Lung infection | Oral | - | W | 1 | [56] |  |
| 52 | ***Cirsium dender* Friis **** [Asteraceae]** | H | Daji umba'o(Sh) | Common cold | Oral | 1 | W | 1 | [68] |  |
| 53 | ***Cirsium englerianum* O. Hoffm. ** [Asteraceae]** | H | Yahyakusheshilie  (Am), Bur (Me) | Influenza virus, Respiratory tract problem | Oral, Nasal | 4 | W | 2 | [42], [86] |  |
| 54 | *Cissampelos mucronata* A.Rich. [Menispermaceae] | Cl | Shamtit (Me) | Respiratory tract problem | oral | - | W | 1 | [42] |  |
| 55 | *Cissampelos pareira* L. [Menispermaceae] | Cl | Gud (KA) | Flu | Oral | 1 | W | 1 | [87] |  |
| 56 | *Cissus quadrangularis* L. [Vitaceae] | Cl | _ | lung infection | Oral, topical | - | W | 1 | [56] |  |
| 57 | *Citrus aurantifolia* (Christm) Swingle [Rutaceae] | T/S | Lomi (Am), Tutto (Ko) | Cough (2), Flu | Oral | 4,8 | WC | 3 | [39], [61], [88] |  |
| 58 | *Citrus lemon* (L.) Burm. f. [Rutaceae] | S | Lomi (Am), Lomae (Ge) | Cough (3), Common cold, Atshma | Oral | 1,4,9 | C | 5 | [52] , [66], [89], [90]., [91] |  |
| 59 | *Citrus medic*a L. [Rutaceae] | S | Tiringo (Am) | Cold | Oral | - | W | 1 | [63] |  |
| 60 | *Clausena anisate* (Willd.) Benth [Rutaceae] | S | Ermic'o(Sh) | Common cold | External | 3 | WC | 1 | [68] |  |
| 61 | *Clematis hirsuta* Perr & Guill [Ranunculaceae] | Cl/H | Azo Hareg (Gu) | Common cold, Cough | Nasal, Oral | 3,4 | W | 2 | [92], [93] |  |
| 62 | *Clerodendrum myricoides* (Hochst.) R. Br. Ex Vatke [Verbnaceae] | S | Misirichi (Am), Marasisa(Or) | Common cold, Dry cough, Asthma, Cough, Cold/Qilensa/ | Oral, Nasal | 1,2,4,6 | W | 4 | [58],[63], [51], [94] |  |
| 63 | *Coffea arabica* L. [Rubiaceae] | S | Bunna (Am) | Asthma,Common cold | Oral | 1 | C | 3 | [58], [84], [86] |  |
| 64 | *Commelina imberbis* Ehrenb. Ex Hask [Commelinaceae] | H | _ | Asthma | NA | - | W | 1 | [95] |  |
| 65 | *Conyza stricta* Willd. [Asteraceae] | H |  | Cough | Oral | 4 | W | 1 | [76] |  |
| 66 | *Corandrium sativum* L. [Apiaceae] | H | Debo (Ka) | Cough | NA | - | C | 1 | [60] |  |
| 67 | *Croton dichogamus* Pax [Euphorbiaceae] | S | Makuffa (Or) | Cold/ Qilensa | NA | - | W | 1 | [63] |  |
| 68 | *Croton macrostachyus* Del. [Euphorbiaceae] | T | Shomo(Sh) | Common cold, Asthma,Pneumonia | External, Oral | 1,3 | WC | 3 | [68], [83], [96] |  |
| 69 | *Croton schimperianus* Muell. Arg. [Euphorbiaceae] | S | Makafta(Or) | Cold /Qilensa | Csm | - | W | 1 | [63] |  |
| 70 | *Cucumis dipsaceus* Ehrenb. ex Spach [Cucurbitaceae] | H/Cl | Basu baqula (Sd) | Cough, Common cold | Fumigaton | 1,9 | W | 2 | [77], [97] |  |
| 71 | *Cucumis ficifolius* A. Rich. [Cucurbitaceae] | H | Waagereechcho (Ke), Yemidir Embuy (Am) | Cough, Dry Cough, Asthma, Pneumonia | Oral | 3,4,8 | W | 3 | [46], [89], [98] |  |
| 72 | *Cucumis sativus* L [Cucurbitaceae] | H | Basubaqula | Pneumonia, cough | Oral | 4 | C | 1 | [59] |  |
| 73 | *Cuscuta epithymum* L. [Convolvulaceae] | Cl |  | Asthma | Oral | 4 | W | 1 | [58] |  |
| 74 | *Cymbopogon citrates* (DC.) Stapf. [Poaceae] | H | Xajsaara (Or),  Tej sar (Am) | Cough (2), pneumonia, Influenza | Oral, Nasal, Fumigation | 1,2,5 | C | 3 | [37], [46], [71] |  |
| 75 | *Datura Stramonium* L. [Solanaceae] | H | Manjii (Or) | Asthma , Cough (2) | Nasal, Oral | 3,5 | W | 2 | [58], [80] |  |
| 76 | *Diospyros abyssinica* (Hiern) F. White [Ebenaceae] | S | Lookoo (Or) | Pneumonia | NA | - | W | 1 | [73] |  |
| 77 | *Dovyalis abyssinica* (A. Rich.) Warb. [Flacourtiaceae] | S | Koshim (Am) | Asthma | oral | 2 | WC | 1 | [81] |  |
| 78 | *Dregea schimperi* (Decne.) Bullock [Apocynaceae] | Cl | - | Asthma | Oral | 1,6 | W | 1 | [14] |  |
| 79 | *Echinops hispidus* Fresen. [Asteraceae] | H |  | Pneumonia | Oral | 8 | W | 1 | [76] |  |
| 80 | ***Echinops kebericho* Mesfin. ** [Asteraceae]** | H | Kebericho (Am), K'ebero(Sh) | Pneumonia, Common cold (4), Cough (2) | Oral, Nasal, Inhale | 1,2,4,5,7 | C/semi W | 9 | [58], [61], [65], [68],[72], [73], [74] , [87] |  |
| 81 | *Echinops macrochaetus* Fresen. [Asteraceae] | H | Qoree-harree (Or) | Pneumonia | NA | - | W | 1 | [73] |  |
| 82 | *Ekebergia capensis* Sparm. [Meliaceae] | T | - | lung TB | oral | 1 | W | 1 | [58] |  |
| 83 | *Ensete ventricosum* (Welw.) Cheesman [Musaceae] | T | Wutto (Ka) | Cough | NA | - | C | 1 | [60] |  |
| 84 | ***Erythrina brucei* Schweint*. ***  [Fabaceae]** | T | Wolensu (Or) | Pneumonia, Cough | oral | 4 | W | 2 | [58], [73] |  |
| 85 | *Eucalyptus camaldulensis* Dehnh. [Myrtaceae] | T | Barzzaafiya (Or), Nech bahairzaf (Am) | Cough, Common cold | Oral, Inhale | 2 | C | 2 | [37], [99] |  |
| 86 | *Eucalyptus globulus* Labill [Myrtaceae] | T | Bahirzaf (Am), Antakirt (Gu) | Cough (13), Common cold (11), Pneumonia, Respiratory problems, Breathing difficulties, Flu, Asthma | Inhale | 2,5,7 | WC | 27 | [14],[58], [46], [83] |  |
| 87 | *Euphorbia abyssinica* Gmel. [Euphorbiaceae] | T | Kolankul (Ti) | Cough | Oral | 1 | W | 1 | [62] |  |
| 88 | *Euphorbia schizacantha* Pax [Euphorbiaceae] | H | Dhetungayda | Cough | Oral | 11 | W | 1 | [61] |  |
| 89 | *Fagonia schweinfurthi*i Hadidi [Zygophyllaceae] | S | - | Lung infection | Oral, body wash | - | NA | 1 | [56] |  |
| 90 | *Ferula communis* L. [Apiaceae] | H | Dog (Or) | Cough (2) | Oral | 1,5 | W | 2 | [71], [93] |  |
| 91 | *Ficus carica* L. [Moraceae] | S | - | Asthma | oral | 3 | W | 1 | [58] |  |
| 92 | *Foeniculum vulgare* Mill*.* [Apiaceae] | H | Ensilal (Am), Harashi(Sh) | Cough (2), Asthma, Common cold | Oral | 1 | C/semi W | 4 | [68],[74] , [86] |  |
| 93 | *Galinsoga parviflora* L. [Asteraceae] | H | Kundo berbere | Common cold (Qufa | Oral | 1,6 | W | 1 | [51] |  |
| 94 | *Gnidia stenophylla* Gilg [Thymaelaceae] | H | Harmala Tiqishu (Or) | Cold /Qilensa | Oral | 6 | W | 1 | [63] |  |
| 95 | ***Gomphocarpus purpurascens* A. Rich. ** [Asclepiadaceae]** | S | Ari-Yuyo (Or) | Pneumonia | Oral, Nasal | 2 | W | 1 | [83] |  |
| 96 | *Grewia erythraea* Schweinf [Tiliaceae] | S | - | Flue | Inhale | - | W | 1 | [56] |  |
| 97 | *Grewia ferruginea* Hochst ex A. Rich [Tiliaceae] | S | Ogomdii (Ge) | Cough (2), Asthma | Oral | 6,11 | W | 3 | [52] , [75], [84] |  |
| 98 | *Guizota abyssinica* (L. f.) Cass. [Asteraceae] | H/S | Nug (Am) | Cough (3), Dry cough, Common cold (2), Asthma, | Oral | 1,8 | C | 4 | [14], [25], [72], [91] |  |
| 99 | *Hallea rubrostipulata* (K. Schum.)J.-F. Leroy [Rubiaceae] | T | Oppo(Sh) | Common cold | Oral | 1 | W | 1 | [68] |  |
| 100 | *Helianthus annuus* L. [Asteraceae] | H | Suf (A) | Cough, Common cold | Oral | 1 | C | 1 | [14] |  |
| 101 | *Heliotropium steudneri* Vatke [Boraginaceae] | S | Kibo (KW) | Chronic Cough | Oral | 11 | W | 1 | [87] |  |
| 102 | *Heteromorpha arborescens* (Spreng.) Cham. & Schlecht. [Apiaceae] | S | Al-Hanqaa | Shivering and unable to breath(Cuma’a) | Oral | 3 | W | 1 | [100] |  |
| 103 | *Hordeum vulgare* L. [Poaceae] | H |  | Cough | Oral | 11 | C | 1 | [101] |  |
| 104 | *Hypoestes forskaolii* (Vahl) R. Br. [Acanthaceae] | H | Omorutta (Kam), Ciikkicho(Si) | Cough (2) | Inhale | 5 | W | 2 | [39], [102] |  |
| 105 | Impatiens ethiopica Grey- Wilson [Balsaminaceae] | H | Insosla | Cough | Oral | 3 | WC | *1* | [81] |  |
| 106 | *Indigofera amorphoides* Jaub.& Spach [Fabaceae] | H | Gurbi adi (Or) | Lung disease /Sombe | Oral | 1 | W | 1 | [63] |  |
| 107 | *Indigofera oblongifolia* Forsk. [Fabaceae] | H | - | Lung Infection | Oral | - | W | 1 | [56] |  |
| 108 | *Indigofera spicata* Forssk. [Fabaceae] | H | Gimay, shersherit, sherit, shamtit (Me) | Cough | oral | - | W | 1 | [42] |  |
| 109 | ***Inula confertiflora* A. Rich. ** [Asteraceae]** | S | Woynagift | Asthma, Common cold, Coughing | Oral | 1 | W | 1 | [7] |  |
| 110 | *Jasminum grandiflorum* L [Oleaceae] | Cl | Tembelel | Common cold | Oral, Nasal | 2 | W | 1 | [103] |  |
| 111 | *Jasmiuum abyssinicum* Hochst. ex DC. [Oleaceae] | Cl |  | Cough | Nasal | 5 | W | 1 | [76] |  |
| 112 | *Juniperus procera* Hochst. ex Endl. [Cupressaceae] | H | Yehabesha- tsid | Cough | Fumigation or inhalation | 2 | WC | 1 | [69] |  |
| 113 | *Justicia schimperiana* (Hochst. Ex Nees) T.Anders. [Acanthaceae] | S | Smiza (Am), Xumunga (Ha) | Common cold, Cough, asthma, pneumonia | Nasal, inhale | 2,7 | C | 2 | [46], [86] |  |
| 114 | ***Kalanchoe petitiana* A. Rich **[Crassulaceae]** | H | Andawula (Andiqui) | Common cold | Nasal | 5 | W | 1 | [103] |  |
| 115 | *Kanahala laniflora* (Forssk.) R. Br [Asclepidaceae] | S | Wundiffo (Ge) | Bronchitis, Flu, Asthma | Oral, Inhale | 11 | W | 2 | [52] , [56] |  |
| 116 | *Kleinia squarrosa* Cufod. [Asteraceae] | S | Luqo (Or) | Cold | NA | - | WC | 1 | [63] |  |
| 117 | Lagenaria siceraria (Molina) [Cucurbitaceae] | H | Amham (Ti) | Influenza | Nasal | 2 | WC | 1 | [62] |  |
| 118 | *Laggera crispate* (Vahl) Hepper & Wood [Asteraceae] | H | Keskese (Or) | Asthma | Nasal | 7 | W | 1 | [65] |  |
| 119 | *Laggera integrifolia* Sch. Bip. ex A. Rich [Asteraceae] | H | Gimmie | Common cold | Nasal | 7 | NA | 1 | [98] |  |
| 120 | ***Laggera tomentosa* (Sch. Bip. ex A. Rich.) Oliv. & Hiern ** [Asteraceae]** | H | Keskeso (Or), Nech kese (Am) | Flu, Common cold | Nasal | 7 | W | 2 | [66], [71] |  |
| 121 | *Lawsonia inermis* L. [Lythraceae] | T | Hinaye (Or) | Cough | Oral | 1 | C | 1 | [104] |  |
| 122 | *Lepidium sativum* L. [Brassicaceae] | H | Feto (Am), Shipo(Ka) | Common cold (2), Cough (2) | Oral | 1,3 | C | 4 | [60],[61],[68],[82] |  |
| 123 | ***Leucas acquistylosa* Sebald ** [Lamiaceae]** | H | Qiibatoora (Ha) | Cough, pneumonia | Oral | 4 | W | 1 | [46] |  |
| 124 | *Linum usitatissimum* L. [Linaceae] | H | Shelala (Sd), Telba (Am) | Asthma | NA | 3 | C | 1 | [77] |  |
| 125 | *Lippia adoensis* Hochst. [Verbenaceae] | H | Kosarata(Or), Kusaayee (Ha) | Cough (2) | Oral | 1,4 | C | 2 | [46], [80] |  |
| 126 | *Lippia javanica* (Burm. f.) Spreng. [Verbenaceae] | S | Kusaye (Or) | Cough | Inhale | 9 | W | 1 | [79] |  |
| 127 | *Lobelia giberroa* Hemsl. [Lobeliaceae] | H | Gadirano (Sh) | Common cold | Oral | 1 | W | 1 | [68] |  |
| 128 | *Lycopersicon esculentum* (L.) Mill. [Solanaceae] | H | Timaatima (Or) | Common cold | Oral | 10 | C | 1 | [80] |  |
| 129 | *Lysimachia ruhmeriana* Vatke [Primulaceae] | H | Wosha bashinqa (Kam) | Cough | Oral | - | W | 1 | [39] |  |
| 130 | *Mangifera indica* L. [Anacardiaceae] | T | Mango (Am) | Cough, Asthma | NA | - | C | 1 | [105] |  |
| 131 | *Marubium vulgare* L. [Lamiaceae] | H | - | Chronic cough | oral | - | WC | 1 | [96] |  |
| 132 | *Melilotus suaveolens* Ledeb. [Fabaceae] | H | Cholo, Maniyo (Ka) | Cough, Asthma | Oral, Nasal | 4 | W | 2 | [60], [68] |  |
| 133 | *Mentha spicata* L. [Lamiaceae] | S | Nana (Am) | Cough and cold | Oral | 1 | C | 1 | [71] |  |
| 134 | *Mimusops kummel* Bruce ex A. DC [Sapotaceae] | T | Eshe (Am) | Asthma | Oral | 3,4 | WC | 2 | [86] |  |
| 135 | *Momordica foetida* Schumach [Cucurbitaceae] | Cl/S | Umba'o(Sh), Yubarrae (Ge) | Common cold, Bronchitis (2) | Oral | 3,10 | W | 3 | [52] , [68],[75] |  |
| 136 | *Moringa stenopetala* (E. G. Baker) Cufod [Moringaceae] | T | Aleko , Shiferawu (Am) | Flu, Asthma (2) | Oral, Inhale | 1,5 | WC | 3 | [87], [106] |  |
| 137 | *Myrica salicifolia* Hochst. ex A. Rich. [Myricaceae] | T | Shinet (Am) | Common cold | Nasal | 7 | W | 1 | [86] |  |
| 138 | *Myrtus communis* L. [Myrtaceae] | S | Adasii (Or) | Pneumonia | Nasal | 2 | WC | 1 | [83] |  |
| 139 | *Nicotiana tabacum* L. [Solanaceae] | H | Timbaho(Me) | Respiratory tract problem | Oral, Nasal | - | C | 1 | [42] |  |
| 140 | *Nigella sativa* L. [Ranunuclaceae] | H | Tiqur-azmud (Am), Habsuuda (Or), Gambelata Xagutta (Kam) | Common cold (2), Asthma (3), Bronchial spasm | Nasal, oral, inhale | 2,6,7 | C | 6 | [58], [25], [39], [73], [98], [111] |  |
| 141 | *Ocimum americanum* L. [Lamiaceae] | H |  | Cough | Oral | 1 | W | 1 | [37] |  |
| 142 | *Ocimum basilicum* L. [Lamiaceae] | H | Kafo(Sh), Besobilla (Am) | Common cold, Flu (2), Cough (infant) | Oral | 1,6,10 | C | 4 | [37], [46], [68],[71] |  |
| 143 | *Ocimum gratissimum* L. [Lamiaceae] | S | Damakasee (Or) | Common cold | Oral | 1 | C | 1 | [107] |  |
| 144 | *Ocimum lamifolium* Hochst. ex Benth. [Lamiaceae] | H/S | Damakesse (Am), Damo (Ka) | Cough (9), Common cold (2), Influenza (2), Pneumonia (2) | Oral, Nasal, Inhale | 1,3,6,7,8,11 | WC | 12 | [14], [58], [46], [52] , [60], [65], [66], [67], [49], [70], [75], [105] |  |
| 145 | *Ocimum tenuiflorum* L. [Lamiaceae] | H | - | Cough | Oral | 4 | NA | 1 | [46] |  |
| 146 | *Ocimum urticifolium Roth [Lamiaceae]* | H | Dama kesie (Am), Yelebe fuanfa (Gu), Delibekera (Ke) | Common cold | Oral, Inhale | 1 | C | 3 | [14],[86], [95] |  |
| 147 | *Olea europaea* L subsp. c*uspidata* (Wall. ex G. Don) Cif. [Oleaceae] | T | - | Asthma | Oral | 1 | WC | 1 | [89] |  |
| 148 | *Olinia rochetiana A. Juss. [Oliniaceae]* | T | Noole (Si) | Common cold | Nasal | 7 | W | 1 | [102] |  |
| 149 | *Opuntia ficus-indica* (L.) Mill [Cactaceae] | S | Beles (Ti) | Pneumonia | Oral | 1 | WC | 1 | [78] |  |
| 150 | *Osyris quadripartita* Salzm. ex Decne. [Santalaceae] | S | Tunto (Sd) | Cough | NA | 3 | W | 1 | [77] |  |
| 151 | ***Otostegia integrifolia* Benth. ** [Lamiaceae]** | S | Tunjit (Am) | Lung disease, Common cold | Inhale | 5 | W | 2 | [49], [86] |  |
| 152 | *Passiflora edulis* Sims [Passifloraceae] | Cl | - | Pneumonia | Nasal | 3 | WC | 1 | [92] |  |
| 153 | *Pavetta gardeniifolia* A. Rich. [Rubiaceae] | S | Wari ampi | Common cold | Inhale | - | W | 1 | [82] |  |
| 154 | *Pavonia burchellii* (DC.) Dyer. [Malvaceae] | S | - | Cough | oral | 8 | W | 1 | [89] |  |
| 155 | *Pentas shimperiana* (Rich) Vatke [Rubiaceae] | H | - | Cough | NA | - | W | 1 | [58] |  |
| 156 | *Peperomia retusa* (L.f.) A. Dietr. [Piperaceae] | H | Gargiyo(Sh), Gongiji(Sh) | Common cold | Oral | 1 | W | 1 | [68] |  |
| 157 | *Phytolocca dodecandra* L ‘Hert [Phytolaccaceae] | H | Andoodee | Sinus | Oral | 8 | W | 1 | [108] |  |
| 158 | *Piper capense* [Piperaceae] | H | Turfo(Sh) | Common cold | Oral | 6 | WC | 1 | [68] |  |
| 159 | *Piper nigrum* L. [Piperaceae] | Cl | Kundo berbere | Common cold | Oral | 9 | C | 1 | [64] |  |
| 160 | *Piper umbellatum* [Piperaceae] | H | Turfo(Sh) | Common cold | Oral | 6 | W | 1 | [68] |  |
| 161 | ***Pittosporum abyssinicum* Del. ** [Pittosporaceae]** | S | Shollo(Ka) | Cough | NA | - | W | 1 | [60] |  |
| 162 | *Pittosporum viridiflorum Sims [Pittosporaceae]* | T | Ulaga (Gu) | Coughing, Pneumonia (Sinbabie) | Oral | 4 | W | 1 | [14] |  |
| 163 | *Plantago lanceolata* L. [Acanthaceae] | H | - | Cough | Oral | 4 | W | 1 | [58] |  |
| 164 | *Plectranthus barbatus* Andrews [Lamiaceae] | H | Yet'o(Sh) | Common cold | Oral | 1 | W | 1 | [68] |  |
| 165 | *Plectranthus cylindraceus* Hochst. ex. Benth [Lamiaceae] | H |  | Common cold | Dermal, nasal | 7 | W | 1 | [76] |  |
| 166 | *Plumbago zeylanicum* L. [Plumbaginaceae] | H | Amira | Chronic cough (2), Asthma | Oral | 1,9 | W | 2 | [93], [109] |  |
| 167 | *Podocarpus falcatus* (Thunb) R. Br. ex Mirb. [Podocarpaceae] | T | Zigba | Cough, Asthma | Oral | 1,4 | C | 2 | [14], [84] |  |
| 168 | *Polygala obtusissima* Hochst. Ex Chod. [Polygalaceae] | H | Calmala | Common cold, Flu, Asthma | Inhale | 7 | W | 2 | [117], [56] |  |
| 169 | *Portulaca olerace*a L. subsp. *oleracea* [Portulacaceae] | H | Merere Haree, Kentela | Cough (2) | Oral | 10 | W | 2 | [58], [110] |  |
| 170 | *Premna schimperi* Engl. [Lamiaceae] | S | Udo (Sd) | Cough | NA | 2 | W | 1 | [77] |  |
| 171 | *Punica granatum* L. [Lythraceae] | S | - | Cough | Nasal | 5 | C | 1 | [58] |  |
| 172 | ***Pycnostachys abyssinica *** [Lamiaceae]** | S | Ye'iro(Sh) | Common cold | External | 2 | W | 1 | [68] |  |
| 173 | *Pycnostachys eminii* Gurke [Lamiaceae] | H | Bok'alekako(Sh) | Common cold | External | 2 | W | 1 | [68] |  |
| 174 | *Pycnostachys meyeri* Gurke [Lamiaceae] | H | Bok'alekako(Sh) | Common cold | External | 2 | W | 1 | [68] |  |
| 175 | *Ranunculus multifidus* Forssk. [Ranunculaceae] | H | Kertassa | Asthma (Asm) | Oral | 6 | W | 1 | [51] |  |
| 176 | *Rhus natalensis* Bernh. ex C.Krauss [Anacardiaceae] | S | - | Cough | Oral | 2 | W | 1 | [61] |  |
| 177 | *Ricinus communis* L. [Euphorbiaceae] | S | Gulo (Amh) | Cough | NA | 12 | WC | 1 | [77] |  |
| 178 | *Ritchiea albersii* Gilg [Capparidaceae] | T | Dalsach kularit (Me) | Cough, Respiratory tract problem | Oral | 4 | W | 2 | [42], [108] |  |
| 179 | *Rotheca myricoides* (Hochst.) Steane [Lamiaceae] | S | Madhisisa | Cough | Oral | 1 | W | 1 | [59] |  |
| 180 | *Rubia cordifolia* L. [Rubiaceae] | H/Cl | Enchibir (Am), Qaccabba (Or) | Cough (8), Asthma (2) | Oral | 1,3,6 | W | 9 | [58], [46], [49], [71], [84], [87], [86] |  |
| 181 | *Rubus steudneri* Schweinf [Rosaceae] | H | Agogota (Or) | Cough | Oral | 1 | W | 1 | [71] |  |
| 182 | *Rumex abyssinicus* Jacq. [Polygonaceae] | H | Mekimeko (A) | Pharyngitis | Oral | 9 | W | 2 | [58], [49] |  |
| 183 | *Rumex nervosus* Vahl. [Polygonaceae] | S | Ambacho | common cold | Oral | 1 | W | 1 | [81] |  |
| 184 | *Ruta chalepensis* L. [Rutaceae] | H | Chena adam (Ti), Sunkurtaa (Sd) | Cough (9), Common cold (4), Lung infection | Oral | 1,3,4,8 | C | 12 | [58], [25], [59], [112] |  |
| 185 | *Saccharum officinarum* L. [Poaceae] | H/S | Shankora (Or) | Common cold (2), Cough | Oral, Inhale | 5,10 | C | 3 | [58], [38], [80] |  |
| 186 | *Salvadora persica* L. [Salvadoreaceae] | S | Mero | Flu | Oral | 11 | W | 1 | [87] |  |
| 187 | *Salvia shimperi* Benth. [Lamiaceae] | H | Haremajiwa | Cough | NA | - | W | 1 | [95] |  |
| 188 | *Satureja abyssinica* (Benth.) Briq. [Lamiaceae] | H | Korsa qufa (Or) | Common cold/ Qufa | Oral | 1 | W | 1 | [63] |  |
| 189 | *Schinus molle* L. [Anacardiaceae] | T | Tikurberb ere, Kundoberberie | Asthma, Common cold, Cough | Oral, Nasal | 7,10 | C | 3 | [84], [86], [99] |  |
| 190 | *Securidaca longepdunculata* Fres [Polygalaceae] | S | - | Lung disease | oral | 3 | WC | 1 | [58] |  |
| 191 | *Seddera hirsute* Dammer ex Hall. f. [Convolvulaceae] | S | - | Asthma | Oral | - | W | 1 | [56] |  |
| 192 | *Senecio hadiensis* Forssk. [Asteraceae] | Cl | Walgabbissa | Shivering and unable to breath (Cuma’a) | Oral | 6 | WC | 1 | [100] |  |
| 193 | *Senna italic* Mill. [Fabaceae] | H | Fitii (Or) | Cough (2) | Nasal | 7 | W | 2 | [80], [95] |  |
| 194 | *Senna septemtrionalis* (Viv.) Irwin & Barneby [Fabaceae] | S | _ | Cough | Oral | 4 | W | 1 | [84] |  |
| 195 | *Sida rhombifola* L. [Malvaceae] | S | Chifrig (A) | Cough | oral | 3 | W | 1 | [58] |  |
| 196 | *Sida schimperiana* Hochst. ex A. Rich. [Malvaceae] | S | Chifrig (A) | Cough (2), Influenza | Oral, Inhale | 2,8 | W | 2 | [61], [49] |  |
| 197 | *Solanecio angulatus* (Vahl) C. Jeffrey [Asteraceae] | H | Jinaras (Or) | Pneumonia | NA | - | W | 1 | [73] |  |
| 198 | ***Solanecio gigas (Vatke)* C. *Jeffrey *** [Asteraceae]** | S | Dombirako(Sh) | Common cold | Oral | 1 | W | 1 | [68] |  |
| 199 | *Solanum aculeatissimum* Jacq. [Solanaceae] | S | Haanja Borbodho (Si) | Asthma | Oral | 1 | W | 1 | [102] |  |
| 200 | *Solanum incanum* L. [Solanaceae] | H/S | Borbodicho, Tilik embuway (Am) | Asthma, Cough, Pneumonia | Oral, nasal | 3,4,12 | W | 3 | [77], [92], [109] |  |
| 201 | *Solanum macrocarpon* Poir. [Solanaceae] | H | - | Cough | Oral | 10 | WC | 1 | [58] |  |
| 202 | *Solanum marginatum* L. f. [Solanaceae] | S/H | Aby ungule (Ti) | Cough | Oral | 3 | W | 2 | [89], [112] |  |
| 203 | *Solanum nigram* L. [Solanaceae] | H | Dame’e (Ha) | Cough | Oral | 1 | W | 1 | [46] |  |
| 204 | *Solanum schimperianum* Hochst. ex A. Rich. [Solanaceae] | S |  | Common cold | Topical | 5, 7 | W | 1 | [101] |  |
| 205 | *Spilanthes uliginosa* [Asteraceae] | H | - | Asthma | NA | - | W | 1 | [58] |  |
| 206 | *Steganotaenia araliacea* Hochst. ex A. Rich. [Apiaceae] | T | Afretoo(Or) | Pneumonia | NA | - | WC | 1 | [73] |  |
| 207 | *Stephania abyssinica* (Dillon & A. Rich.) Walp. [Mensipermaceae] | H | Hidda kalaalaa (Or) | Common cold (2), Pneumonia | Oral, Inhale | 2 | W | 3 | [38], [39], [79] |  |
| 208 | *Tamarindus indica* L. [Fabaceae] | T | _ | Lung Infection | Oral, nasal | - | W | 1 | [56] |  |
| 209 | ***Taverniera abyssinica* A. Rich ** [Fabaceae]** | S | Dingete (Or) | Whooping cough | Oral | 1 | W | 1 | [65] |  |
| 210 | *Tephrosia elata* Deflers [Fabaceae] | S | Kashabach (Me) | Respiratory tract problem | Oral | - | W | 1 | [42] |  |
| 211 | *Tephrosia villosa* (L.) [Fabaceae] | H | Kashabach, zangalech (Me) | Respiratory tract problem | Oral | - | W | 1 | [42] |  |
| 212 | *Thunbergia alata* Sims. [Acanthaceae] | C | Hareg (Or) | Cough | Oral | 3 | W | 1 | [71] |  |
| 213 | *Thymus schimperi* Ronniger [Lamiaceae] | H | Tosign (Am) | Cough (2), Common cold, Whooping cough | Oral | 1,4 | WC | 4 | [68], [70], [95], [113] |  |
| 214 | ***Thymus serrulatus* Hoechst ex. Benth ** [Lamiaceae]** | H | Tosign (Am) | Cough, Respiratory problems | Oral | 1,6 | W | 2 | [58], [67] |  |
| 215 | *Thymus vulgaris* L. [Lamiaceae] | H | Toshine (Am, Gu) | Continuous cough | Oral | 4 | WC | 1 | [114] |  |
| 216 | *Tribulus cistoides* L. [Zygophyllaceae] | H | Mugro (KA, KW) | Flu | Oral | 3 | W | 1 | [87] |  |
| 217 | *Trigonella foenum-graecu* [Fabaceae] | H | Absh (Am) | Dry cough | Oral | 1 | C | 1 | [72] |  |
| 218 | *Triticum aestivum* L. [Poaceae] | H |  | Cough | Oral | 1 | C | 1 | [101] |  |
| 219 | *Uvaria leptocladon* Oliv. [Anonaceae] | T | Zebko (Ka), Chochum (KW) | Cough | Oral | 11 | W | 1 | [87] |  |
| 220 | *Verbascum sinaiticum* Benth. | H | Daba Keded (Am) | Asthma | Oral | 3 | W |  | [81] |  |
| 221 | *Verbascum stelurum* Murb [Scrophulariaceae] | H | _ | Cough | oral | - | W | 1 | [58] |  |
| 222 | *Verbena officinalis* L. [Verbenaceae] | H | Atuch (Or), Modolita(Kam) | Cough (3) | Oral | 6 | WC | 3 | [58],[39], [71] |  |
| 223 | *Vitis vinifera* L. [Vitaceae] | Cl | - | Cough | oral | 1 | C | 1 | [58] |  |
| 224 | *Warburgia ugandensis* Sprague [Canellaceae] | T | Befit (Or) | Cough (3), Asthma | Inhale, Nasal | 5 | W | 4 | [58], [49], [106] |  |
| 225 | *Withania somnifera* (L) Dunal [Solanaceae] | S | Giziewa (Am) | Common cold, Cough, Pneumonia | Oral, Inhale | 1,5,8 | WC | 3 | [49], [86], [115] |  |
| 226 | *Ximenia americana* L. [Oleaceae] | S/T | Mekela (Ka), Waljoweljo (Kw), Hudaa(Or) | Flu, Lung abscess | Oral | 3,6 | W | 2 | [87], [88] |  |
| 227 | *Zanthoxylum chalybeum* Engl. [Rutaceae] | T | Gedai (Ma) | Common cold | Oral | - | W | 1 | [82] |  |
| 228 | *Zehneria scabra* [Cucurbitaceae] | S | Hafaflo(Ti) | Cough | NA | 2 | W | 1 | [116] |  |
| 229 | *Zingiber officinale* Rosc. [Zingebiraceae] | H | Jinjibila (Am), Gamchalech (Me) | Cough (10), Common cold (8), Asthma, Influenza, Respiratory tract problem with cough | Oral | 1,3,9 | C/semi W | 14 | [14], [58], [42] |  |
